# Effect of metformin on nonalcoholic fatty liver based on meta-analysis and network pharmacology

**DOI:** 10.1097/MD.0000000000031437

**Published:** 2022-10-28

**Authors:** Yuanshe Huang, Xiaodong Wang, Chen Yan, Chen Li, Lidan Zhang, Lai Zhang, E Liang, Tianlei Liu, Jingxin Mao

**Affiliations:** a AnShun University, Guizhou Anshun, China; b College of Pharmaceutical Sciences, Southwest University, Chongqing, China; c Chongqing Medical and Pharmaceutical College, Chongqing, China; d An Shun City People’s Hospital, Anshun, China; e Department of Biology, Chemistry, Pharmacy, Free University of Berlin, Berlin, Germany.

**Keywords:** candidate drug, meta-analysis, metformin, network pharmacology, nonalcoholic fatty liver disease, therapeutic effect

## Abstract

Whether metformin is related to nonalcoholic fatty liver disease (NAFLD) is controversial. Our aim was to investigate the relationship between metformin and NAFLD that may predict the metformin potential of these lesions and new prevention strategies in NAFLD patients.

**Methods:**

The meta-analysis was analyzed by Revman 5.3 softwares systematically searched for works published through July 29, 2022. Network pharmacology research based on databases, Cytoscape 3.7.1 software and R software respectively.

**Results:**

The following variables were associated with metformin in NAFLD patients: decreased of alanine aminotransferase (ALT) level (mean difference [MD] = −10.84, 95% confidence interval [CI] = −21.85 to 0.16, *P* = .05); decreased of aspartate amino transferase (AST) level (MD = −4.82, 95% CI = −9.33 to −0.30, *P* = .04); decreased of triglyceride (TG) level (MD = −0.17, 95% CI = −0.26 to −0.08, *P* = .0002); decreased of total cholesterol (TC) level (MD = −0.29, 95% CI = −0.47 to −0.10, *P* = .003); decreased of insulin resistance (IR) level (MD = −0.42, 95% CI = −0.82 to −0.02, *P* = .04). In addition, body mass index (BMI) (MD = −0.65, 95% CI = −1.46 to 0.16, *P* = .12) had no association with metformin in NAFLD patients. 181 metformin targets and 868 NAFLD disease targets were interaction analyzed, 15 core targets of metformin for the treatment of NAFLD were obtained. The effect of metformin on NAFLD mainly related to cytoplasm and protein binding, NAFLD, hepatitis B, pathway in cancer, toll like receptor signaling pathway and type 2 diabetes mellitus (T2DM). The proteins of hypoxia inducible factor-1 (HIF1A), nuclear factor erythroid 2-related factor (NFE2L2), nitric oxide synthase 3 (NOS3), nuclear receptor subfamily 3 group C member 1 (NR3C1), PI3K catalytic subunit alpha (PIK3CA), and silencing information regulator 2 related enzyme 1 (SIRT1) may the core targets of metformin for the treatment of NAFLD.

**Conclusion:**

Metformin might be a candidate drug for the treatment of NAFLD which exhibits therapeutic effect on NAFLD patients associated with ALT, AST, TG, TC and IR while was not correlated with BMI. HIF1A, NFE2L2, NOS3, NR3C1, PIK3CA, and SIRT1 might be core targets of metformin for the treatment of NAFLD.

## 1. Introduction

nonalcoholic fatty liver disease (NAFLD) has become the most common chronic liver disease in the world and the incidence rate of NAFLD has been increasing in recent years.^[1]^ NAFLD is characterized by abnormal accumulation of triglycerides in hepatocytes, not due to secondary hepatic steatosis caused by excessive alcohol consumption or other reasons.^[2]^ NAFLD is usually divided into 2 histological categories including nonalcoholic fatty liver (NAFL) and nonalcoholic steatohepatitis (NASH) which cause of liver disease with prevalence estimates ranging from 25% to 45%.^[3]^ NAFLD includes a series of progressive liver diseases, ranging from simple steatosis to NASH, with fibrosis usually developing into cirrhosis and hepatocellular carcinoma (HCC).^[4]^ Diet and exercise therapy are the cornerstone for NAFLD, but there is still no ideal treatment drug at present.

Previous studies have shown that obesity, insulin resistance (IR), dyslipidemia, abnormal glucose metabolism and hypertension are risk factors of NAFLD.^[5]^ Liver lipid metabolism disorder, IR, the role of cytokines, increased expression of cytochrome P450 (CYP2E1, CYP4A), oxidative stress, lipid peroxidation, immune response, genetic factors and other related factors are all involved in the pathogenesis of NAFLD.^[6]^ Therefore, improving IR and adjusting the balance of glucose and lipid metabolism may be an important measure for the prevention and treatment of NAFLD. In addition, NAFLD is not only related to morbidity and mortality of liver, but also with the increased risk of other diseases such as chronic kidney disease and colorectal carcinoma.^[7]^

Many researchers have conducted a series of animal experiments and clinical studies on the efficacy of metformin on NAFLD.^[8]^ Metformin is an insulin sensitizer which may improve insulin sensitivity by increasing the binding of peripheral insulin and insulin receptor, increasing the clearance of blood sugar and improving insulin sensitivity.^[9]^ Furthermore, the gastrointestinal discomfort which caused by metformin can reduce the food intake and body weight of obese patients.^[10]^ Previous clinical studies have shown that metformin may improve the liver enzymes involved in lipid metabolism in patients with NAFLD and regulate the expression of liver fat hormones and adipokines.^[11]^ However, it remains controversial including studies on the ineffectiveness of metformin to NAFLD have also been reported.^[12]^ In addition, the effect and mechanism of metformin on NAFL is unclear. Therefore, the aim of the meta-analysis and network pharmacology research was to further assess the effects of metformin on NAFLD and figured out the most likely mechanism of metformin on NAFLD.

## 2. Methods

We followed the methods of Jingxin Mao et al 2020 and 2022, respectively.^[13,14]^

### 2.1. Search strategy

The applicable posted articles including PubMed, Embase databases, and ISI Web of Science databases were used to investigated until July 29, 2022. The following keywords were used in searching: “metformin” OR “met” AND “nonalcoholic fatty liver disease OR NAFLD OR nonalcoholic fatty liver NAFL OR nonalcoholic steatohepatitis OR NASH OR fatty liver” AND clinic trial OR treatment OR relationship. Relevant articles have been used to expand the search scope, and all retrieved studies, evaluations and convention abstracts had been retrieved by using the computer. If more than one posted studies describe the same population, we extract solely the most entire or latest one immediately. Two authors independently accomplished the decision procedure and finally resolved the differences via discussion.

### 2.2. Selection criteria

The selection/determination strategy used the following criteria: English language studies; Randomized controlled/managed clinical trial or potential or retrospective original research or cohort that explored the impact of metformin on NAFLD; To diagnosed of NAFLD used to be primarily based on standardized ultrasound examination or pathologic examination, The relevant records together with alanine aminotransferase (ALT), aspartate amino transferase (AST), total cholesterol (TC), triglyceride (TG), body mass index (BMI) and homeostasis model assessment of insulin resistance index (HOMA-IR) had been all analyzed statistically.

The exclusion standards had been adapted to exclude studies from meta-analysis as following: Non-English language studies; Reviews, case reports, editorials, letters to editors, congress abstracts, commentaries, exercise guidelines, conferences or convention records; Insufficient data (for instance: less than 30 participants in the study); Single diagnostic criteria for NAFLD without imaging examination (for instance: only using fatty liver index or serum liver enzyme index); Studying length past 20 years, Autoimmune, drug-induced liver disease/disorder or liver damage.

### 2.3. Data extraction

Two authors abstracted the following data from the included articles: first author, country, publication years, study design, case number, diagnosis method, covariate adjustment, met dose of treatment group and Newcastle-Ottawa quality assessment scale (NOS). Any disagreements were resolved by a third investigator. The NOS was finally been used to assess the quality of the study.

### 2.4. Explore the anti-NAFLD target of metformin

Validated and predicted targets of metformin were obtained from the SuperPred (https://prediction.charite.de/index.php) and SwissTarget Prediction (http://www.swisstargetprediction.ch/) databases. Using “nonalcoholic fatty liver disease” and “nonalcoholic hepatic disease” as keywords to search for NAFLD-related targets in CTD database (http://ctdbase.org/). The metformin targets retrieved above were mapped with NAFLD disease targets to obtain potential targets of metformin in the treatment of NAFLD, which were imported into the Uniprot database (https://www.uniprot.org/) and converted into corresponding gene names. Eliminate non-human targets in preparation for the subsequent topological analysis of protein protein interaction (PPI) networks.

### 2.5. PPI network construction, analysis and core gene screening

Import the gene names obtained in 2.4 into the STRING database, the species is limited to homo sapiens, the confidence is set to > 0.17, and the others are the default settings, create a PPI network map and save the TSV format file. The above results were imported into the cytohubba plugin of Cytoscape 3.7.1 software, and the maximal clique centrality (MCC) calculation method was selected to screen out the core target genes.

### 2.6. Biological function and pathway enrichment analysis of common drug-disease targets

The clusterProfiler R software was used to conduct GO functional enrichment analysis and KEGG pathway enrichment analysis on the potential targets of metformin in the treatment of NAFLD. *P* < .05 indicated that the difference was statistically significant. The above results were visualized using the R language gg-Plot2 package.

### 2.7. Component-target molecular docking validation

Using the Zinc database (http://zinc.docking.org/) to search the molecular structure of metformin and download its MOL2 format. Core target genes were screened in section 2.5 through the PDB database (http://www.rcsb.org/) to obtain the suitable protein structure and download it in PDB format. With AutoDockTools software, the molecular structure of metformin was optimized, and the core gene protein structure was dewatered and hydrogenated, and the molecule and protein storage format was converted to “pdbqt” format. Using Vina software for molecular docking, collect binding energy scores, and finally use PyMOL software for visual analysis.

### 2.8. Ethics statement

The meta-analysis and network pharmacology research were strictly followed the ethical approval that referenced publication articles which related to human care, handling, sampling and administration procedures should be approved by ethics committee.

### 2.9. Patient and public involvement

Patients and the public were not involved in the design, conduct, reporting, or dissemination plans of this research.

### 2.10. Statistical analysis

Using Ravman Manager softwares (version 5.3, Cochrane Collaboration) for statistical evaluation and analysis. The magnitude of the impact of each studying was utilized to calculated by the mean difference (MD) and the odds ratio (OR) of 95% confidence interval (CI) respectively. A *P* value <.05 was once regarded statistically significant. Furthermore, the heterogeneity was quantified the use of the *Q* test and the *I*^2^ statistic. While *P* > .1 and *I*^2^ < 50%, a fixed-effect model was finally used otherwise a random-effect model was finally applied. In addition, Begg funnel plots were used to calculated for viable publication bias.

## 3. Results

**Figure 1.**
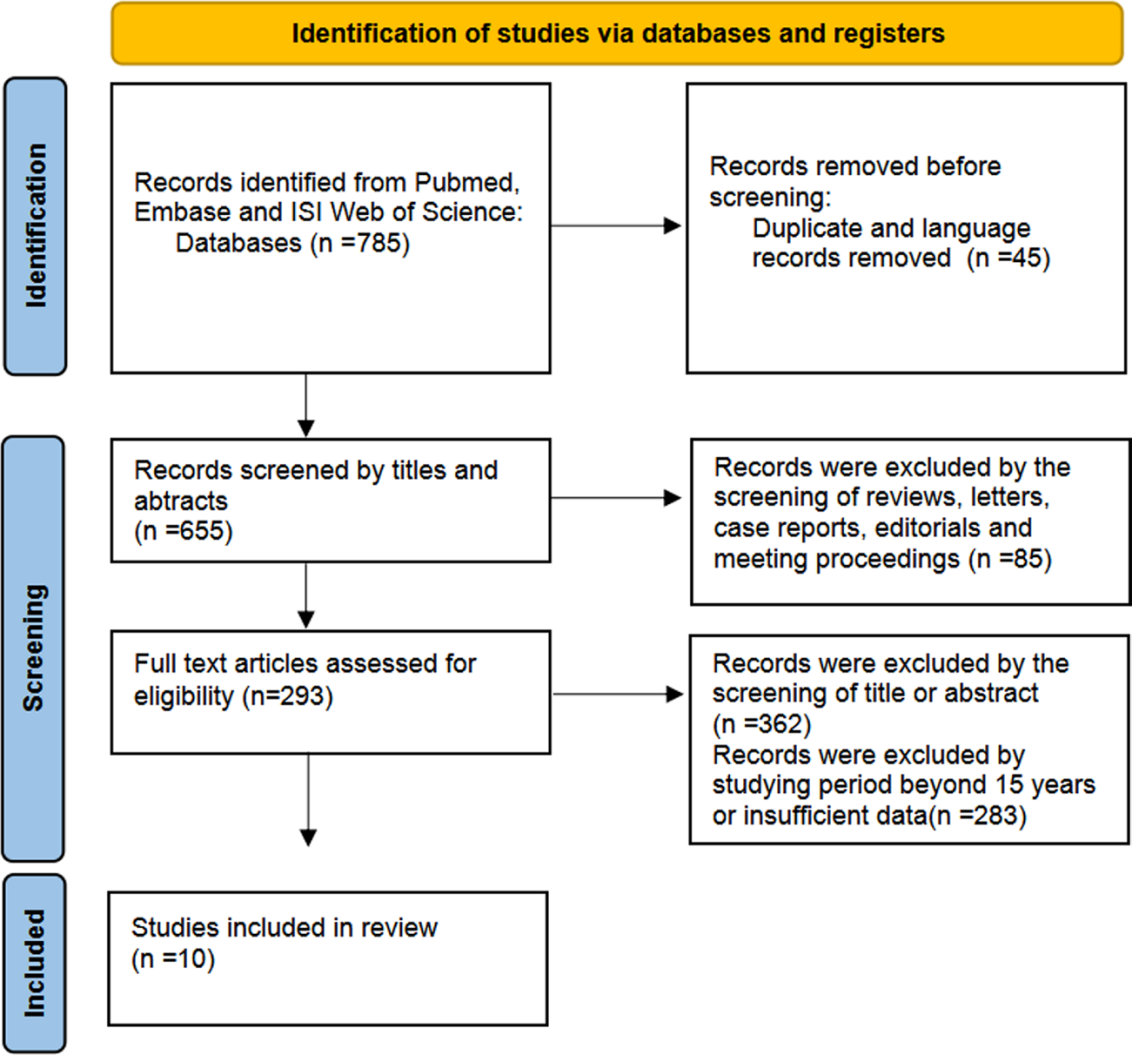
Flow chart of the study selection process.

After searching, a total of 10 studies which met our selection criteria were finally included in the present meta-analysis. The selection flowchart of study was presented in Figure 1. The basic characteristics of the studies was included in Table 1. In addition, the network pharmacology of metformin on NAFLD was presented in the manuscript. Through network pharmacology data mining, 181 metformin targets and 868 NAFLD disease targets were obtained, and 38 potential targets of metformin in the treatment of NAFLD were obtained by mapping. 15 core target interaction relationships were obtained after PPI network calculation by MCC method. GO enrichment analysis and KEGG pathway analysis further elucidate the functional information of biological processes, cellular components closely with cytoplasm and protein binding, molecular functions pathways that mainly related to NAFLD, hepatitis B, pathway in cancer, toll like receptor signaling pathway and type 2 diabetes mellitus. Molecular docking results showed that the binding energies of metformin and core targets were all < 0 kJ mol^−1^, and the binding energies of hypoxia inducible factor-1 (HIF1A), nuclear factor erythroid 2-related factor (NFE2L2), nitric oxide synthase 3 (NOS3), nuclear receptor subfamily 3 group C member 1 (NR3C1), PI3K catalytic subunit alpha (PK3CA), and silencing information regulator 2 related enzyme 1 (SIRT1) and metformin were <−5 kJ mol^−1^ significantly.

**Table 1:** Basic characteristics of included studies on the association between hypothyroidism and NAFLD.

First author	Country	Publication yrs	Study design	Case number	Duration (mo)	Diagnosis method	Covariate adjustment	Met dose of treatment group	NOS
Feng^[15]^	China	2018	Prospective, randomized trial	93	24	Ultrasonography	Age, sex, AST, ALT, insulin resistance, blood glucose, body weight, BMI, TG, TC, HOMA- IR, HDL, LDL	250 mg first wk, 500 mg second wk, and 1000 mg third wk	8
Garinis^[16]^	Italy	2010	Prospective study	50	6	Ultrasonographic	Age, sex, AST, ALT, insulin resistance, glucose, BMI, TG, TC, HOMA- IR, HDL, LDL	1000 mg/d	7
Handzlik- Orlik^[17]^	Poland	2018	Open-label, randomized study	42	6	Ultrasound	AST, ALT, Insulin, BMI, TG, TC, HOMA- IR, GGTP	500 mg first wk, 2000 mg second wk	8
Haukeland ^[18]^	Norway	2009	Proof-of-concept study	44	6	Biopsy	Weight, AST, ALT, insulin, BMI, TG, TC, HOMA-IR, free fatty acid	500–3000 mg/d	7
Idilman^[19]^	Turkey	2008	Prospective study	129	6	Biochemical, radiological and histological criteria	Age, gender, weight, AST, ALT, BMI, TG, TC, HOMA, body fat content, plasma glucose	850 mg/d	9
Loomba^[20]^	American	2009	Open-label study	28	12	MRI and CT, imaging and liver biopsy	Age, gender, weight, AST, ALT, BMI, TG, TC, total bilirubin, LDL, HDL	500 mg once daily, first wk, 500 mg twice daily, second wk to 3 wk, 1000 mg twice daily	8
Nar^[21]^	Turkey	2008	Prospective study	34	6	Ultrasonographic	Age, gender, AST, ALT, BMI, TG, TC, hypertension, glucose, LDL, HDL, leptin	850–1700 mg/d	7
Nobili^[22]^	Italy	2008	Open-label, observational pilot study	57	24	Biopsy and ultrasonographic	Age, gender, AST, ALT, γ-GGT, BMI, TG, TC, HOMA	1.5 g/d	8
Sofer^[23]^	Israel	2011	Single-center study	63	4	Ultrasonography	Age, gender, BMI, AST, ALT, TG, HOMA-IR, HOMA-β, HDL, LDL	850–1700 mg/d	8
Uygun^[24]^	Turkey	2004	Randomized controlled study	36	6	Ultrasonography	Age, gender, AST, ALT, BMI, TG, TC, glucose， index of insulin resistance	850 mg/d	7

ALT = alanine aminotransferase, AST = aspartate amino transferase, HDL = high density lipoprotein, HOMA = homeostasis model assessment, HOMA-IR = insulin resistance index, LDL = low density lipoprotein, NAFLD = nonalcoholic fatty liver disease, NOS = Newcastle-Ottawa quality assessment scale, TG = triglyceride, TC = total cholesterol, γ-GGT = γ- glutamyl transferase.

### 3.1. Effect of metformin on NAFLD patients (Table 2 )

#### 3.1.1. Effect of metformin on ALT level

**Figure 2.**
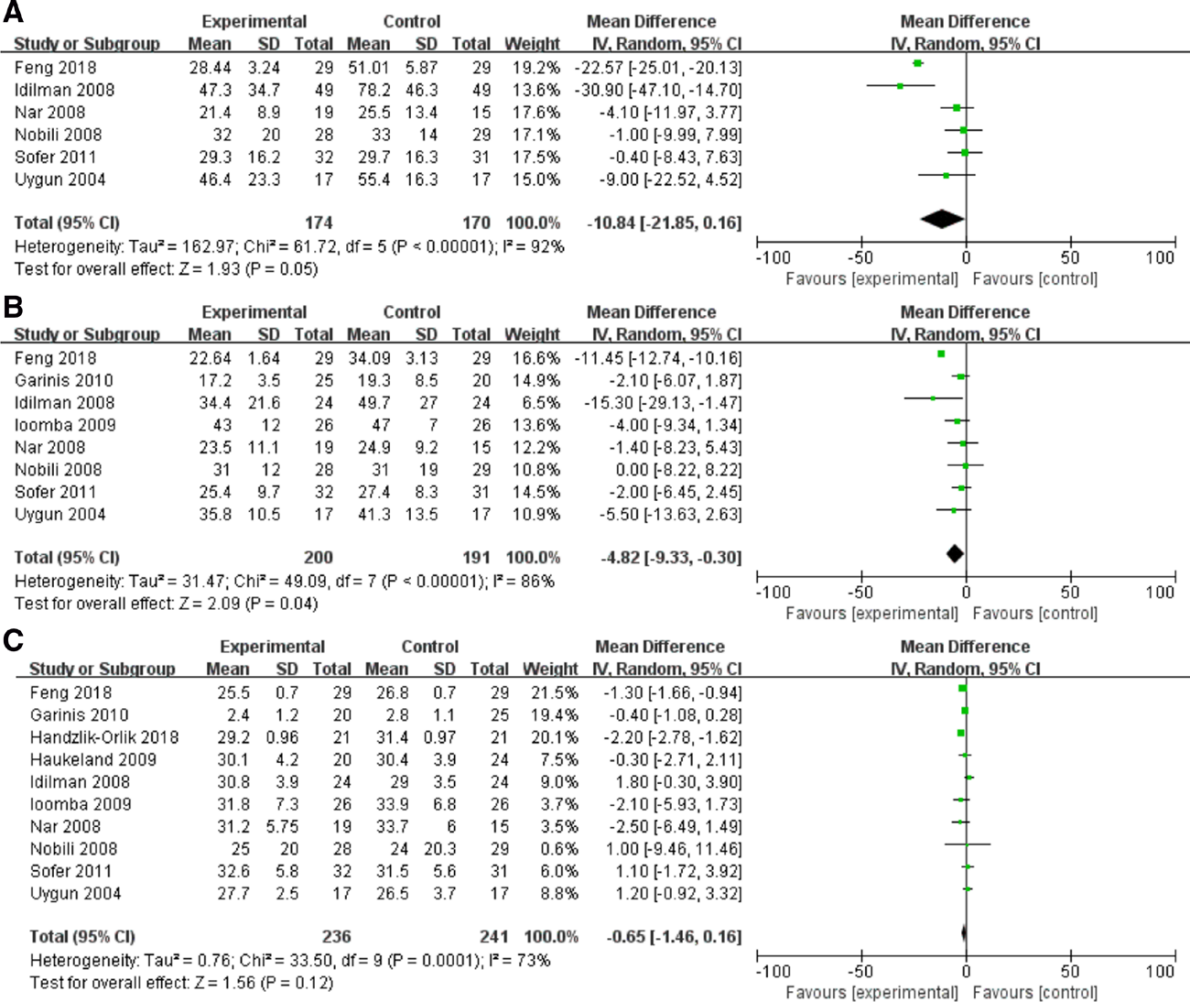
Forest plots of the effect of metformin on (A) ALT, (B) AST and (C) BMI level. ALT = alanine aminotransferase, AST = aspartate amino transferase, BMI = body mass index.

A random-effects model and input continuous data were selected using inverse variance method to calculated (*P* < .00001, *I*^2^ = 92%). The results indicated that a significant association was existed between metformin and ALT, metformin may decrease the level of ALT in NAFLD patients (MD = −10.84, 95% CI = −21.85 to 0.16, *P* = .05) (Fig. 2A).

**Table 2:** Effect of metformin on NAFLD patients.

Related indicators	MD	95% CI	*P* value
ALT	−10.84	−21.85 to 0.16	.05
AST	−4.82	−9.33 to −0.30	.04
BMI	−0.65	−1.46 to 0.16	.12
TG	−0.17	−0.26 to −0.08	.0002
TC	−0.29	−0.47 to −0.10	.003
IR	−0.42	−0.82 to −0.02	.04

CI = confidence interval, MD = mean difference, NAFLD = nonalcoholic fatty liver disease.

#### 3.1.2. Effect of metformin on AST level

Total of 8 studies were investigated for the relationship between AST and NAFLD patients. A random-effects model and input continuous data were selected using inverse variance method to calculated (*P* < .00001, *I*^2^ = 86%). It was revealed that metformin could decreased the level of AST in NAFLD patients (MD = −4.82, 95% CI = −9.33 to −0.30, *P* = .04) (Fig. 2B).

#### 3.1.3. Effect of metformin on BMi

Ten included studies were explored for the effect of metformin on BMI in the NAFLD patients. A random-effects model was utilized to analyze the data (*P* = .0001, *I*^2^ = 73%). It was demonstrated that metformin was not related to BMI in patients with NAFLD (MD = −0.65, 95% CI = −1.46 to 0.16, *P* = .12) (Fig. 2C).

#### 3.1.4. Effect of metformin on TG level

**Figure 3.**
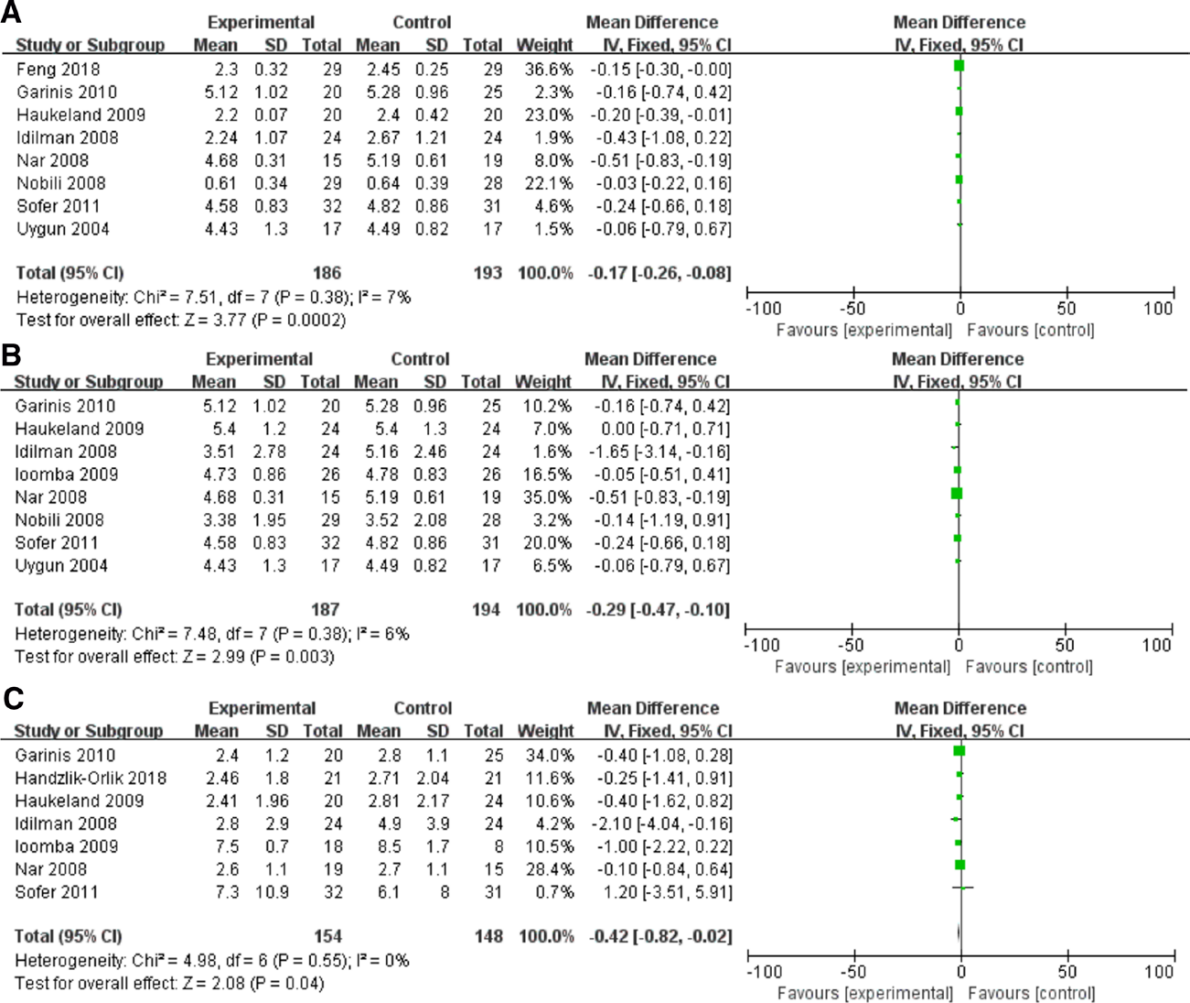
Forest plots of the effect of metformin on (A) TG, (B) TC and (C) IR level. IR = insulin resistance, TG = triglyceride, TC = total cholesterol.

Eight studies were analyzed for the correlation between metformin and the level of TG in NAFLD patients. A fixed-effects model was used to analyze the data (*I*^2^ = 7%). In the meta-analysis, the unit of measurement has been unified as mmol/L. (TG: 88.5 mg/dL = 1 mmol/L). It was demonstrated that metformin was significantly relatively related to TG in NAFLD patients (MD = −0.17, 95% CI = −0.26 to −0.08, *P* = .0002) (Fig. 3A).

#### 3.1.5. Effect of metformin on TC level

In the meta-analysis, the unit of measurement has been unified as mmol/L (TC: 100 mg/dL = 2.6 mmol/L). A random-effects model was utilized to analyze the data (*I*^2^ = 82%). Six included studies were evaluated for the relationship between metformin and TC in NAFLD patients. It was revealed that metformin can significantly improve the high level of TC in NAFLD patients (MD = −0.29, 95% CI = −0.47 to −0.10, *P* = .003) (Fig. 3B).

#### 3.1.6. Effect of metformin on IR

A fixed-effects model was utilized to analyze the data (*I*^2^ = 0%). Seven included studies were investigated for the relationship between metformin and IR in NAFLD patients, and the IR was evaluated by HOMA-IR. It was revealed that metformin was closely associated with HOMA-IR in NAFLD patients which means metformin could significantly reduce the level of IR (MD = −0.42, 95% CI = −0.82 to −0.02, *P* = .04) (Fig. 3C).

#### 3.1.7. Publication bias and sensitivity analysis

**Figure 4.**
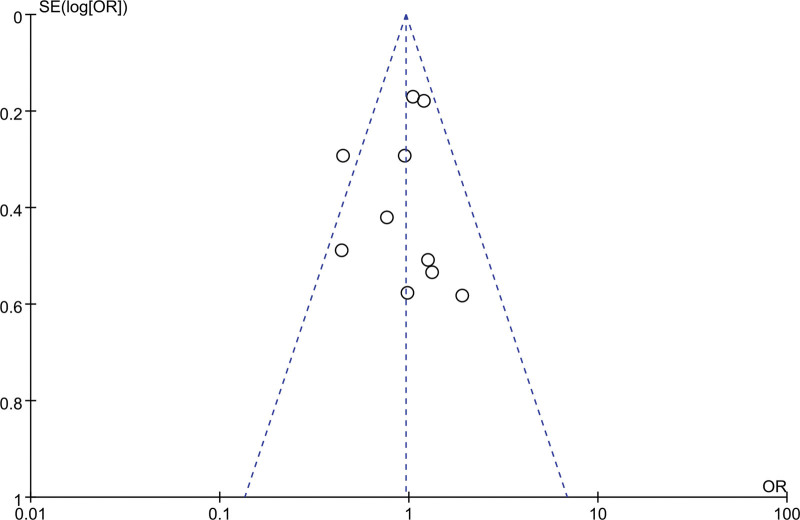
Funnel plots for publication bias analysis of the included articles.

Cochrane funnel plot was used to explore the publication bias. No obvious asymmetric distribution exhibits in Figure 4 which indicating that there was no publication bias.

### 3.2. Network pharmacology of metformin on NAFLd

#### 3.2.1. Metformin anti-NAFLD targets

**Figure 5.**
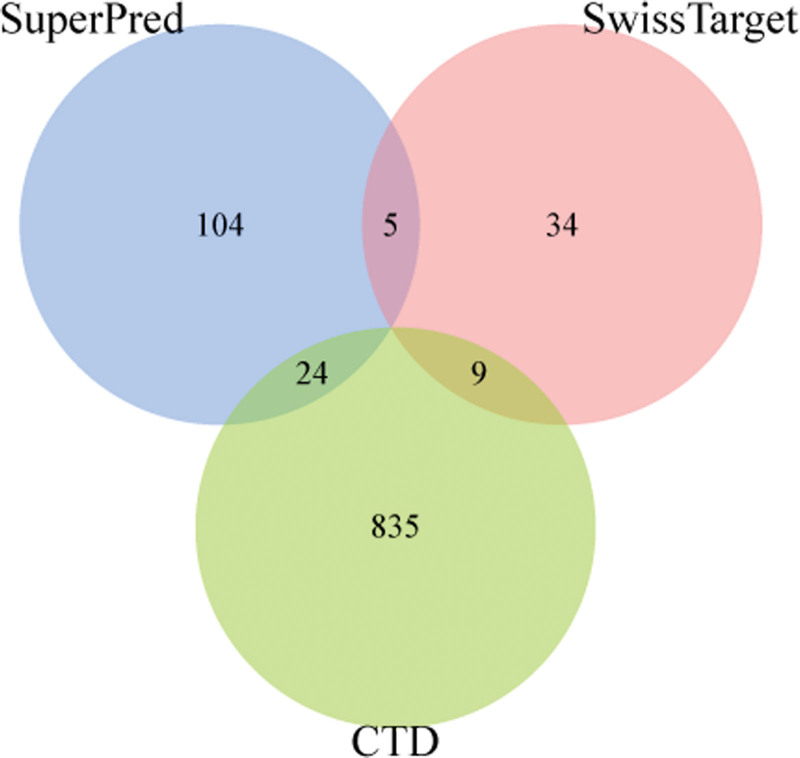
The venn analysis of metformin on NAFLD. NAFLD = Nonalcoholic fatty liver disease.

A total of 868 genes were collected as NAFLD disease targets through CTD database. 133 verified targets and 48 predicted targets of metformin were retrieved using SuperPred database and SwissTargetPrediction database, and 38 intersection targets were obtained after mapping with NAFLD targets point (Table 3 and Fig. 5), predicted to be the potential target of metformin for the treatment of NAFLD.

**Table 3:** NAFLD related targets from metformin.

Gene names	Entry	Protein names	Length (bp)
PLAU	P00749	Urokinase-type plasminogen activator	431
ADORA1	P30542	Adenosine receptor A1	326
METAP2	P50579	Methionine aminopeptidase 2	478
CA1	P00915	Carbonic anhydrase 1	261
TYMP	P19971	Thymidine phosphorylase	482
VDR	P11473	Vitamin D3 receptor	427
FGR	P09769	Tyrosine-protein kinase	529
RPS6KA5	O75582	Ribosomal protein S6 kinase alpha-5	802
ACACA	Q13085	Acetyl-CoA carboxylase 1	2346
IKBKB	O14920	Inhibitor of nuclear factor kappa-B kinase	756
PIK3CA	P42336	Phosphatidylinositol 4,5-bisphosphate 3-kinase catalytic subunit alpha isoform	1068
NR4A1	P22736	Nuclear receptor subfamily 4 group A member 1	598
DPP9	Q86TI2	Dipeptidyl peptidase 9	863
HIF1A	Q16665	Hypoxia-inducible factor 1-alpha	826
NFE2L2	Q16236	Nuclear factor erythroid 2-related factor 2	605
NR3C1	P04150	Glucocorticoid receptor	777
SIRT3	Q9NTG7	NAD-dependent protein deacetylase sirtuin-3, mitochondrial	399
AURKB	Q96GD4	Aurora kinase B	344
GLS	O94925	Glutaminase kidney isoform, mitochondrial	669
MAP4K2	Q12851	Mitogen-activated protein kinase kinase kinase kinase 2	820
CCR2	P41597	C-C chemokine receptor type 2	374
PIK3R1	P27986	Phosphatidylinositol 3-kinase regulatory subunit alpha	724
NFKB1	P19838	Nuclear factor NF-kappa-B p105 subunit	968
SIRT1	Q96EB6	NAD-dependent protein deacetylase sirtuin-1	747
TDO2	P48775	Tryptophan 2,3-dioxygenase	406
NR1I2	O75469	Nuclear receptor subfamily 1 group I member 2	434
APEX1	P27695	DNA-(apurinic or apyrimidinic site) endonuclease	318
CYP3A4	P08684	Cytochrome P450	503
RPS6KB1	P23443	Ribosomal protein S6 kinase beta-1	525
NOS2	P35228	Nitric oxide synthase, inducible	1153
NOS1	P29475	Nitric oxide synthase, brain	1434
SLC47A1	Q96FL8	Multidrug and toxin extrusion protein 1	570
NOS3	P29474	Nitric oxide synthase, endothelial	1203
INMT	O95050	Indolethylamine N-methyltransferase	263
SLC22A1	O15245	Solute carrier family 22 member 1	554
HRH2	P25021	Histamine H2 receptor	359
ESR2	Q92731	Estrogen receptor beta	530
ACHE	P22303	Acetylcholinesterase	614

NAFLD = nonalcoholic fatty liver disease.

#### 3.2.2. Biofunction and pathway enrichment analysis of metformin-potential targets of NAFLD

**Figure 6.**
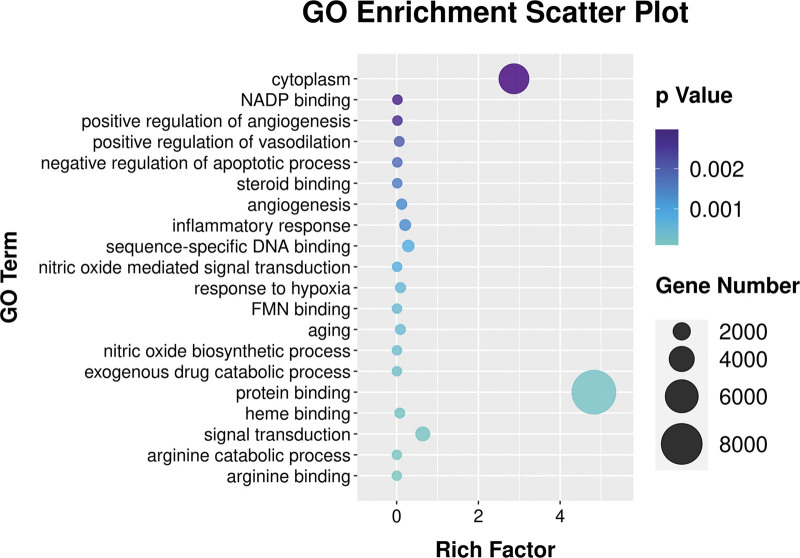
GO enrichment analysis of metformin on NAFLD. NAFLD = Nonalcoholic fatty liver disease.

**Figure 7.**
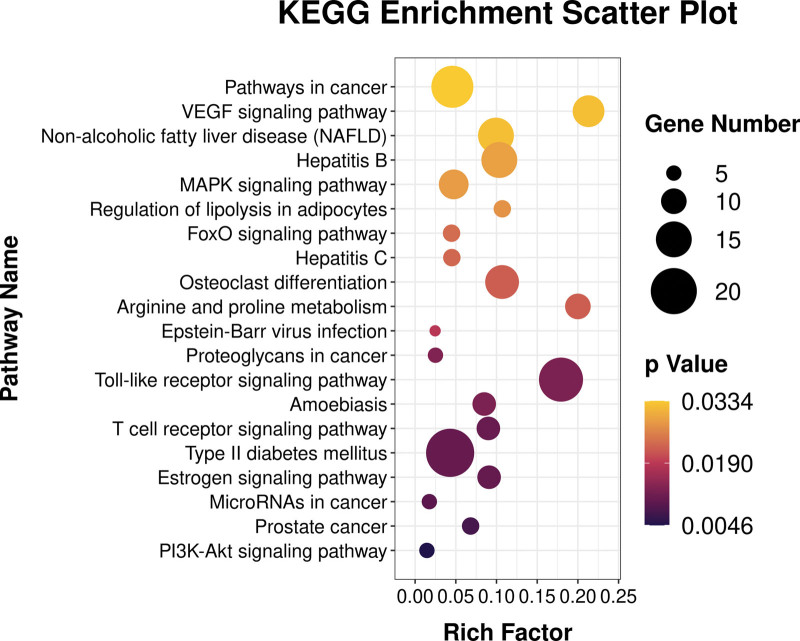
KEGG pathway enrichment analysis of metformin on NAFLD. NAFLD = Nonalcoholic fatty liver disease.

The clusterProfiler R software was used to analyze the biological processes and molecular functions of potential targets involved in NAFLD, and to explored the possible molecular mechanism of metformin in the treatment of NAFLD. The GO function enrichment results were sorted by significance (*P* < .05), and the top 20 items in various analyses were selected for R software visualization (Fig. 6). The potential targets of metformin in the treatment of NAFLD mainly involve cytoplasm, NADP binding, positive regulation of angiogenesis, positive regulation of vasodilation, negative regulation of apoptotic process, steroid binding, angiogenesis, inflammatory response, sequence-specific DNA binding, nitric oxide mediated signal transduction, response to hypoxia, FMN binding, aging, nitric oxide biosynthetic process, exogenous drug catabolic process, protein binding, heme binding, signal transduction, arginine catabolic process, arginine binding. KEGG pathway enrichment analysis screened the top 20 signaling pathways for visual analysis (*P* < .05) (Fig. 7). The NAFLD signaling pathways involved mainly including pathways in cancer, VEGF signaling pathway, non-alcoholic fatty liver disease (NAFLD), Hepatitis B, MAPK signaling pathway, regulation of lipolysis in adipocytes, FoxO signaling pathway, Hepatitis C, Osteoclast differentiation, Arginine and proline metabolism, Epstein-Barr virus infection, Proteoglycans in cancer, Toll-like receptor signaling pathway, Amoebiasis, T cell receptor signaling pathway, Type II diabetes mellitus, Estrogen signaling pathway, MicroRNAs in cancer, Prostate cancer, PI3K-Akt signaling pathway.

#### 3.2.3. The results of PPI network construction, analysis and core gene screening

**Figure 8.**
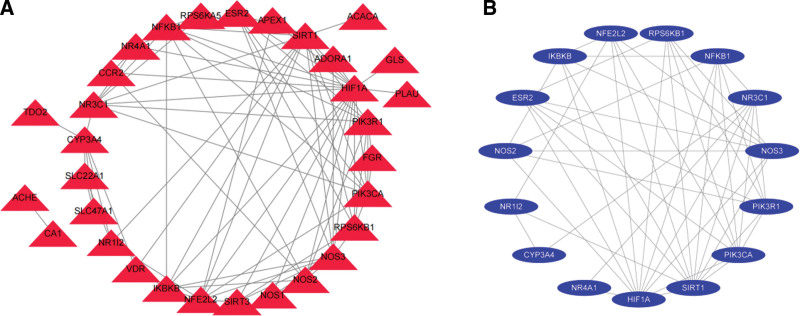
PPI network analysis diagram of potential targets of metformin on NAFLD. NAFLD = nonalcoholic fatty liver disease, PPI = protein protein interaction.

Import the potential targets mined in metformin anti-NAFLD targets into the STRING database, and save the PPI network map and TSV format files. The nodes in the figure represent proteins, and the edges represent the interaction between proteins, which fully reflects the complexity of the molecular mechanism of metformin in the treatment of NAFLD. The nodes in the figure represent proteins, and the edges represent the interaction between proteins, which fully reflects the complexity of the molecular mechanism of metformin in the treatment of NAFLD (Fig. 8A). Import the above files into the cytohubba plug-in, and use the MCC method to calculate and screen out HIF1A, SIRT1, NOS3, PIK3CA, NR3C1, NFE2L2, PIK3R1, NFKB1, IKBKB, NOS2, RPS6KB1, CYP3A4, ESR2, NR1I2, and NR4A1 targets in the core position (Table 4 and Fig. 8B). Its interaction with other proteins is more closely related, and these 15 targets can be predicted to be the key targets of metformin in the treatment of NAFLD.

**Table 4:** Core targets of metformin on NAFLD and their topological characteristics.

Gene names	Targets names	Betweenness centrality	Degree
HIF1A	Hypoxia-inducible factor 1-alpha	0.3527774	18
SIRT1	NAD-dependent protein deacetylase sirtuin-1	0.20641763	14
NOS3	Nitric oxide synthase, endothelial	0.04175313	10
PIK3CA	Phosphatidylinositol 4,5-bisphosphate 3-kinase catalytic subunit alpha isoform	0.05492052	9
NR3C1	Glucocorticoid receptor	0.17371514	8
NFE2L2	Nuclear factor erythroid 2-related factor 2	0.05083879	8
PIK3R1	Phosphatidylinositol 3-kinase regulatory subunit alpha	0.03997753	8
NFKB1	Nuclear factor NF-kappa-B p105 subunit	0.03621829	8
IKBKB	Inhibitor of nuclear factor kappa-B kinase	0.02620307	7
NOS2	Nitric oxide synthase, inducible	0.02237358	7
RPS6KB1	Ribosomal protein S6 kinase beta-1	0.01082576	7
CYP3A4	Cytochrome P450	0.15933485	6
ESR2	Estrogen receptor beta	0.00339315	6
NR1I2	Nuclear receptor subfamily 1 group I member 2	0.07004283	4
NR4A1	Nuclear receptor subfamily 4 group A member 1	0.01549404	4

NAFLD = nonalcoholic fatty liver disease

#### 3.2.4. Component-target molecular docking validation

**Figure 9.**
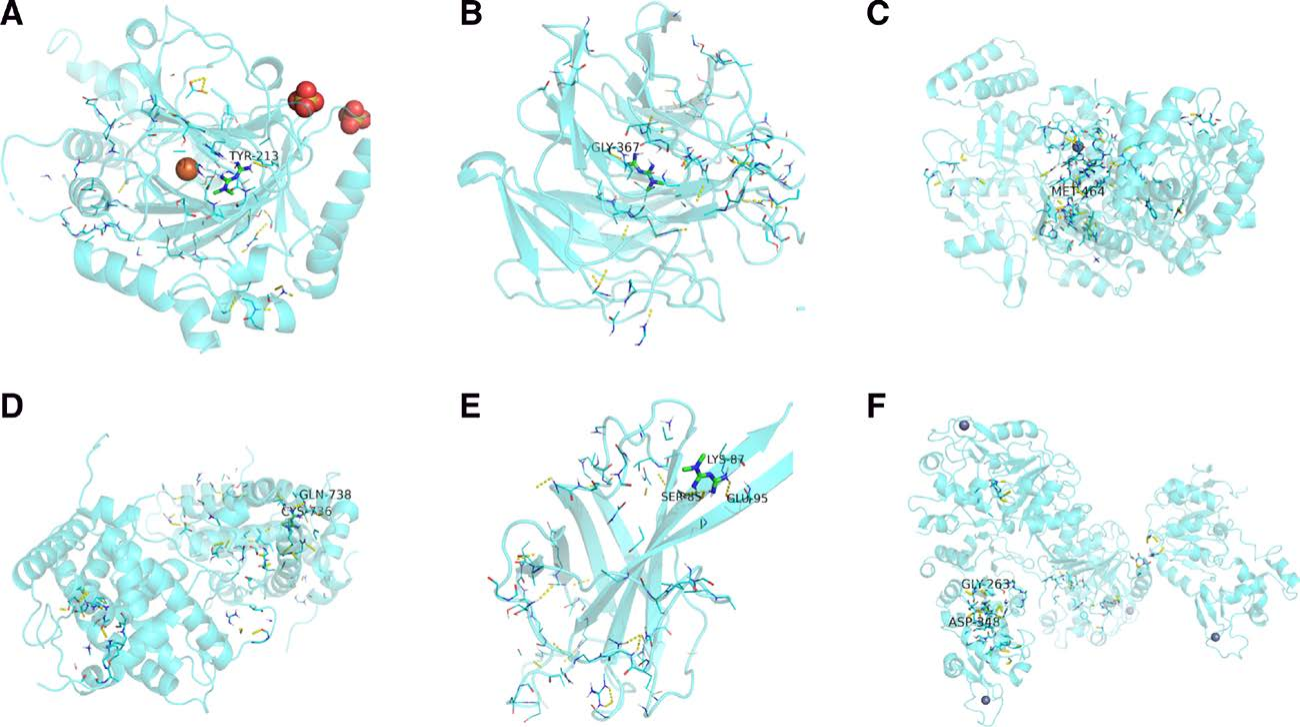
Molecular docking results of metformin on NAFLD with core targets. NAFLD = Nonalcoholic fatty liver disease.

A total of 6 core targets with degree >8 and metformin among the 10 core target genes screened in PPI network were entered into the PDB and Zinc databases, to obtained the molecular structure of metformin and target protein structure respectively. The interaction between proteins and small molecule receptors was simulated by molecular docking technology, and the binding energy values of docking were collected. The low-energy stable conformation between ligand and receptor indicates that there is a greater possibility of interaction between the 2. The binding energies of metformin and core target proteins are all negative, suggesting that ligands and receptors can bind spontaneously. The binding energies of HIF1A, NFE2L2, NOS3, NR3C1, PK3CA, SIRT1 and metformin are <−5 kJ mol^−1^ among them. It was proved that the above targets had stronger binding activity to metformin (Table 5 and Fig. 9).

**Table 5:** The results of molecular docking.

Compound	Target	PDB	Energy (kcal/mol)
metformin	HIF1A	1H2K	−5.0
metformin	SIRT1	4IG9	−5.0
metformin	NOS3	1M9J	−6.4
metformin	PIK3CA	2ENQ	−5.0
metformin	NR3C1	1M2Z	−6.4
metformin	NFE2L2	2F1U	−6.1

PDB = protein data bank.

## 4. Discussion

The incidence of NAFLD and NASH is increasing due to the obesity and diabetes epidemics.^[25]^ It has been demonstrated that the benefits of metformin in inhibiting hepatic gluconeogenesis, improving hepatic fatty acid metabolism (including inhibition of adipose tissue lipolysis), increasing fatty acid oxidation, inhibiting lipogenesis and enhancing insulin sensitivity.^[26]^ Metformin as a treatment for NAFLD/NASH has been partly examined by pilot studies and randomized controlled clinical trials in the past. In addition, previous studies have been reported these beneficial effects of metformin on liver histology in NAFLD/NASH patients to to reduce mortality and fibrosis.^[27,28]^ In addition, Metformin was also suggested to reduce risks for HCC and protect against NASH-related HCC.^[29]^ NAFLD is among the most common causes of liver disease worldwide. Increasing exercise and reasonable planning of diet are all conventional treatments for patients with NAFLD which is expected to reduce weight, but this process takes a long time and is prone to sustained liver injury.^[30]^ Previous studies have shown that NAFLD is closely related to metabolic syndrome including obesity, diabetes, hyperlipidemia and hypertension are risk factors of NAFLD.^[31]^ In addition, some researches has demonstrated that pathophysiological mechanism of these risk factors is IR which exists in more than 90% of NAFLD patients.^[32]^ The pathogenesis of NAFLD is complex and closely related to type 2 diabetes mellitus (T2DM).^[33]^ Generally accepted, it is interpreted as the “two-hit” hypothesis, which means the systemic multiple system damage caused by IR and its secondary disorder of glucose and lipid metabolism.^[34]^ Therefore, correcting the disorder of glucose metabolism and improving IR maybe an important measure to treat NAFLD patients.

Metformin is a recognized the first-line drug to treatment for T2DM. It increases the insulin sensitivity of the peripheral and liver, reduces the production of basic liver glucose, and increases the glucose uptake and utilization of insulin-stimulated peripheral tissues.^[35]^ In recent years, new indications for metformin treatment have appeared, including polycystic ovary syndrome and obesity.^[36]^ Due to NAFLD patients are often accompanied by decreased blood glucose, dyslipidemia, and obesity, metformin may have a beneficial effect on liver steatosis.^[37]^ The exact mode of action of metformin in liver steatosis has not been fully explored, but it may be involved in the destruction of the mitochondrial oxidation process.^[38]^

The present meta-analysis revealed that metformin may has a certain therapeutic effect on NAFLD that short-term (no more that 6 months), low-dose (500 to 3000 mg/d) metformin could ameliorate the levels of ALT, AST, TG and TC in NAFLD patients, and significantly improve HOMA-IR finally. It was showed that metformin can improve the disorder of lipid metabolism, reduce the steatosis and inflammatory response of the liver, and this effect does not depend on the hypoglycemic effect and exists independently.

Transaminase especially ALT and AST considered to be the common indicator for evaluating whether liver cells are damaged.^[39]^ In a variety of liver diseases including viral hepatitis, alcoholic liver disease, drug-induced liver damage, autoimmune hepatitis and other diseases, ALT and AST always exhibit an upward trend.^[40]^ ALT is mainly found in liver cells, while AST is mainly distributed in myocardium.^[41]^ It was reported that ALT and AST can be used as a biomarker to diagnosis of progress of NAFLD even the increased in the normal range.^[42]^ However, in the laboratory examination of early NAFLD patients, the rise of enzyme has no significant specificity, and it is easy to ignore the occurrence of liver injury. In present study, it was found that metformin has closely association with ALT and AST in NAFLD patients. With the increase of metformin dosage and time, both of ALT (MD = −10.84, *P* = .05) and AST (MD = −4.82, *P* = .04) showed a downward trend.

BMI = body weight (kg)/[height (m)^2^], which is a usually research measure used to divide the weight of study participants into broad groups, typically underweight, normal weight, overweight and corpulent classes (obese categories).^[43]^ It was reported that generally the mean or median BMI is about 24 to 27 in Western population-based studies.^[44, 45]^ Therefore, the consequence of adopting the world health organization (WHO) classification is that ~50% or additional of the final adult population may continuously be within the overweight (now preobese) and corpulent classes.

It was also revealed that BMI which related to obese is the most useful predictive factor for the onset of NAFLD which means a poorer prognosis ultimately.^[46]^ In addition, obesity has reduced insulin sensitivity and higher susceptibility to NAFLD, which is also the main reason for the multiple body fatness of NAFLD patients.^[47]^ However, according to our analysis data, metformin was not significantly improve BMI in patients with NAFLD (MD = −0.65, *P* = .12). These relatively conflicting findings between different studies might be related to different characteristics of the patients studied, including sample sizes and proportions of different types of NAFLD. Furthermore, the negligible effect of metformin on weight has been shown before.^[48]^

Eight studies were analyzed for the relationship between metformin and the level of TG in NAFLD patients. The present study has demonstrated that metformin was closely association with TG which exhibits a downtrend in NAFLD patients (MD = −0.17, *P* = .0002). It was revealed that the liver can not only decompose and utilize the stored body fat, but also store the digested and absorbed fat as body fat, which is dynamically balanced in the normal population.^[49]^ However, once this balance is broken, TG is deposited in the liver to form fatty liver in metabolic disorders.^[50]^ In addition, NAFLD is closely related to dyslipidemia and elevated blood sugar. When the liver accumulates fat, its gluconeogenesis strengthens, resulting in increased blood sugar, hyperglycemia can stimulate increased insulin secretion, causing hyperinsulinemia, which further promotes liver synthesis of TG aggravates the lipid deposition of the liver and forms a vicious circle.^[51]^ Hyper TG causes increased release of free fatty acids, which interferes with the binding of insulin to receptors in the surrounding tissues, resulting in insulin resistance.^[52]^

TC refers to the cholesterol contained in various lipoproteins in serum which including he sum of bound cholesterol and free cholesterol.^[53]^ TC is the main component of the cell membrane, therefore the serum concentration of TC can be used as an indicator of lipid metabolism.^[54]^ A large amount of TG synthesized in NAFLD patients with fatty liver may reducing the high-density lipoprotein cholesterol levels and affecting synthesis of high-density lipoprotein cholesterol.^[55]^ When extensively TG is produced in the NAFLD patient’s body, the level of TC may also significantly increased, which is also a sign of severe damage to the patient’s liver.^[56]^ In present study, the effect of metformin on TC (MD = −0.29, *P* = .003) and TG (MD = −0.17, *P* = .002) levels were statistically significant, indicating that metformin can significantly improve the blood lipid levels of patients with NAFLD.

Previous study have proved the closely relationship between NAFLD and IR which indicating NAFLD is consider to be an early marker of IR.^[57]^ Meantime, NAFLD is also a metabolic stress-induced liver injury that related to IR which may causes hepatic steatosis and genetic susceptibility.^[58]^ Furthermore, it was also found that metformin may improves liver function, HOMA-IR in NAFLD patients.^[59]^ In present study, it was revealed that patients with NAFLD have severe IR, which is closely related to the pathogenesis of NAFLD. Furthermore, metformin was effectively on improving IR (MD = −0.42, *P* = .04).

The network pharmacology of metformin on NAFLD results showed that the core targets, significant GO function enrichment and KEGG pathways enrichment. In present study, a total of 181 metformin targets and 868 NAFLD disease targets were interaction analyzed, and 38 potential and 15 core targets of metformin for the treatment of NAFLD were obtained. The effect of metformin on NAFLD mainly related to cytoplasm and protein binding of GO function enrichment respectively, as well as NAFLD, hepatitis B, pathway in cancer, toll like receptor signaling pathway and type 2 diabetes mellitus respectively of KEGG pathways enrichment. It was demonstrated that NAFLD is related to elevated cytoplasmic calcium signaling in hepatocytes^[60]^ which is similar with our predicted GO function enrichment. In addition, sterol regulatory element binding protein has also been reported to play a critical role in NAFLD lipogenesis and correlates with a poor prognosis in NAFLD^[61]^ which is also consistent with our predicted result. Interestingly, the NAFLD and hepatitis B pathway were also obtained in the KEGG pathways enrichment which further proves the correctness of our conjecture and analysis. It was revealed that the co-existence of NAFLD and chronic hepatitis B is common among patients that may synergistically exacerbate liver fibrosis and hepatocellular carcinoma.^[62]^ It was reported that NAFLD-induced lipid accumulation may accelerate tumorigenesis through activation of PI3K. However, the protein kinase Akt is a key factor in hepatic insulin output to glucose.^[63]^ Therefore, pathway in cancer is related to NAFLD. Previous study suggests that intestinal dysbiosis, intestinal barrier dysfunction, and activated toll-like receptor 4 signaling play key roles in the pathogenesis of NAFLD.^[64]^ It was demonstrated that toll like receptor signaling pathway is closely with NAFLD. It has been found that NAFLD is not an isolated disease, but part of a metabolic disorder caused by high energy intake, obesity, sedentary lifestyle, and key IR and T2DM.^[65]^ The prevalence of NAFLD and T2DM has risen markedly because of both of them may share similar risk factors, epidemiology, and pathophysiology. Furthermore, the presence of T2DM significantly increased the odds of NASH and fibrosis compared with NAFLD without T2DM.^[66]^ The above studies confirmed NAFLD is strongly connection with T2DM which is similar with our predicted result. It was reported that the higher the degree value of the target topology analysis, the better the connectivity of this type of targets and the regulation of the entire network.^[67]^ Based on the principle, the proteins of HIF1A, NFE2L2, NOS3, NR3C1, PK3CA, and SIRT1 were considered to be the core targets of metformin for the treatment of NAFLD.

## 5. Conclusion

Taken together, this systematic evaluation and network pharmacology research have shown that metformin could improve biochemical and metabolic characteristics in the progression and the development of NAFLD patients. Furthermore, metformin was related to ALT, AST, TG, TC and IR while was not correlated with BMI in NAFLD patients. HIF1A, NFE2L2, NOS3, NR3C1, PIK3CA and SIRT1 may be the core targets of metformin in the treatment of NAFLD. Therefore, metformin might be a promising drug for the treatment of NAFLD due to its metabolic effect and safety curative effect. These studies have extended the knowledge on the complicated mechanism of NAFLD, and provided the prevention options of NAFLD in patients with metabolic diseases.

## 6. Strengths and limitations of this study

There still exist some limitations in the present meta-analysis and network pharmacology research. Firstly, the heterogeneity is significant in some studies. We attempted to explore the heterogeneity between studies through a random effects model to give a more conservative effect estimate. Secondly, criteria of diagnosis cannot reach uniformity. Due to liver biopsy is not feasible in the general population, some non-standardization methods such as ultrasound, computed tomography, magnetic resonance imaging, and spectroscopy are commonly used to diagnose NAFLD. Thirdly, only 10 suitable studies were included for investigating the effect of metformin on NAFLD patients. Fourth, the manuscript is only based on network pharmacology software and database for prediction without experimental validation. In future studies, large-scale and long-term randomized controlled trials should be conducted in different populations to provide more important evidence. In addition, the experimental validation for predicting key targets of metformin on NAFLD should be carried out at the cellular and animal levels respectively.

## Author contributions

JXM conceived and designed the research. YSH, XDW, and JXM conducted statistical analysis and wrote the paper. LZ, CL, TLL and EL abstracted the total data from the included articles in the meta-analysis. YSH, JXM, XDW, LDZ and LZ completed the data analysis of network pharmacology research. All authors contributed to manuscript revision, read and approved the submitted version.

**Conceptualization:** Jingxin Mao.

**Data curation:** Yuanshe Huang, Xiaodong Wang, Chen Yan, Lidan Zhang, Lai Zhang, Tianlei Liu.

**Formal analysis:** Lai Zhang, Tianlei Liu.

**Funding acquisition:** Yuanshe Huang.

**Investigation:** Yuanshe Huang, Xiaodong Wang, Chen Yan, Chen Li.

**Methodology:** Yuanshe Huang, Xiaodong Wang, Chen Yan, Lidan Zhang, E Liang, Jingxin Mao.

**Resources:** Jingxin Mao.

**Software:** Xiaodong Wang, Chen Li, Lai Zhang, E Liang, Tianlei Liu.

**Supervision:** E Liang.

**Validation:** Chen Yan, Chen Li, Lidan Zhang.

**Visualization:** Yuanshe Huang, Xiaodong Wang, Jingxin Mao.

**Writing – original draft:** Yuanshe Huang.

**Writing – review & editing:** Chen Yan, Jingxin Mao.

## Acknowledgments

The authors would like to thank Prof Min Chen of the College of Pharmaceutical Sciences, Southwest university for helpful discussions on topics related to this work.
